# White Matter Hyperintensities Relate to Basal Ganglia Functional Connectivity and Memory Performance in aMCI and SVMCI

**DOI:** 10.3389/fnins.2019.01204

**Published:** 2019-11-13

**Authors:** Alaka Acharya, Xia Liang, Weiming Tian, Chuanlu Jiang, Ying Han, Liye Yi

**Affiliations:** ^1^School of Life Science and Technology, Harbin Institute of Technology, Harbin, China; ^2^Laboratory for Space Environment and Physical Sciences, Harbin Institute of Technology, Harbin, China; ^3^Department of Neurosurgery, The Second Affiliated Hospital of Harbin Medical University, Harbin, China; ^4^Department of Neurology, XuanWu Hospital, Capital Medical University, Beijing, China; ^5^Center of Alzheimer’s Disease, Beijing Institute for Brain Disorders, Beijing, China; ^6^National Clinical Research Center for Geriatric Disorders, Beijing, China

**Keywords:** subcortical vascular cognitive impairment, amnestic mild cognitive impairment, basal ganglia, functional connectivity, white matter hyperintensity

## Abstract

Cerebral small vessel diseases play a crucial role in both vascular and non-vascular dementias. The location of white matter hyperintensities (WMHs), a neuroimaging marker of cerebral small vessel disease, has been found to vary between different types of dementias, and those in the basal ganglia (BG) have been particularly associated with vascular cognitive impairment (VCI). However, anatomical variation of WMHs across BG nuclei and its effect on brain network dysconnectivity has not been clearly elucidated. The study sample consisted of 40 patients with amnestic mild cognitive impairment (aMCI), 40 with subcortical vascular MCI (SVMCI), and 40 healthy control subjects. We examined the volume of WMH using T2-weighted magnetic resonance imaging. We also assessed the disturbances in BG-cortical communication by measuring resting-state functional connectivity (rsFC) from the functional magnetic resonance imaging signal. WMHs were more pronounced in the SVMCI group particularly in the caudate regions. In SVMCI patients, while higher WMHs in the dorsal caudate correlated with weaker FC with executive control regions and worse immediate recall performance, WMHs in the ventral caudate were associated with weaker FC with anterior default mode regions and worse delayed recall performance. In contrast, in aMCI patients, BG WMHs were not correlated with their changes in functional connectivity changes, which showed weaker connectivity with almost all BG structures, rather than restricting to specific BG subdivisions as observed in the SVMCI group. Our findings demonstrate that heterogeneously distributed BG WMHs are associated with changes in functional network interactions and verbal episodic memory performance only in SVMCI patients, which establishes a link between cerebrovascular-related structural abnormality, functional integrity of BG circuits, and episodic memory impairments in SVMCI, and may reflect a differential role of the cerebrovascular pathology in disrupting network-level communications and cognition between Alzheimer’s and subcortical vascular dementia.

## Introduction

Cerebral small vessel disease (SVD) are considered the primary cause of vascular cognitive impairment (VCI) (Chui, 2007; Pantoni, 2010) and are increasingly recognized to be involved in the etiology of traditionally “non-vascular” dementia of Alzheimer’s disease (AD) (Kalaria and Ihara, 2013; O’Brien and Markus, 2014). Chronic ischemia caused by SVD is often clinically characterized by white matter hyperintensities (WMHs) seen on magnetic resonance imaging (MRI) scans. The clinical relevance of these WM lesions has been observed to be largely regulated by their volume and location (Seo et al., 2010). While evidence has shown that patients with AD exhibit more WMHs in the centrum semiovale, patients with VCI are more likely to have WMHs in the basal ganglia (BG) (Hansen et al., 2015; Banerjee et al., 2017). The BG are a series of subcortical nucleus that can be divided into different divisions, with projections to cortical structures contributing to diverse brain functions (Alexander, 1986; Redgrave et al., 2010; Nelson and Kreitzer, 2014). What has not been tested, however, is whether WMH contents of different BG subdivisions, in the context of their marked anatomical and functional diversities, are differentially vulnerable to VCI.

The development of WMHs has been found to correlate with degeneration of myelin and axons (Scarpelli et al., 1994; Scheltens et al., 1995). With the loss of WM content, one might expect a disruption in functional interactions to the regions with WM lesions. Experimental evidence from both animal and human studies has indeed demonstrated that local WM lesions are related to reductions in functional connectivity (O’Reilly et al., 2013; Langen et al., 2017). Although dysconnectivity of the cortical-BG circuits has been suggested to contribute to cognitive dysfunction in vascular dementia (Cummings, 1994; Looi and Sachdev, 2000), little is known about how functional connectivity of different BG nucleus changes and how location-specific WMHs in the BG affect its functional connections with the cortex.

Episodic memory decline is among the most affected cognitive domains in VCI (Almkvist et al., 1993; Looi and Sachdev, 1999; Buckley, 2006). A growing literature from both neuroimaging and neuropsychological studies suggests involvement of specific BG nucleus in episodic memory processes (Scimeca and Badre, 2012). For example, while the dorsomedial striatum receiving major projections from dorsolateral prefrontal cortices (dLPFC) has been suggested to support episodic memory by promoting efficient encoding of information to later be remembered (O’Reilly and Frank, 2006; Landau et al., 2009; Cools and D’Esposito, 2011), the ventral striatum receiving inputs primarily from the medial prefrontal cortex (mPFC) and limbic structures has been found to be more reliably associated with memory retrieval success (O’Doherty et al., 2004; Tricomi and Fiez, 2009; Cohen and Frank, 2010). However, it is still poorly understood how WM lesions and functional dysconnectivity of BG affect learning and memory performance in VCI.

The goal of this study was to evaluate WMHs and functional network connectivity of different BG subdivisions in patients with subcortical vascular mild cognitive impairment (SVMCI, the most common and homogeneous subtype of VCI) and amnestic MCI (aMCI). We hypothesized that WM lesions may be heterogeneously distributed across different BG structures in both aMCI and SVMCI cohorts but are more severe in SVMCI. We also aimed to investigate how alterations of WMHs and network-level functional interactions of BG nucleus relate to episodic learning and memory deficits. We hypothesized that higher WMHs in specific BG regions in SVMCI would disrupt functional interactions in cortical-BG neurocircuitry in association with abnormal learning and memory performance.

## Materials and Methods

### Demographical Information

One hundred twenty right-handed participants, including 40 SVMCI patients, 40 aMCI patients, and 40 healthy controls participated in this study and were matched on age, sex, and years of education. The MCI patients were recruited from outpatients who visited the memory clinic at the neurology department of XuanWu Hospital, Capital Medical University, Beijing, China. Eligible patients were enrolled if they met clinical criteria for MCI and exclusion criteria as described in our manuscript. Participants with a major diagnostic category of non-neurodegenerative nature, which could otherwise explain cognitive symptoms, were not considered for recruitment. This study was approved by the Medical Research Ethics Committee and Institutional Review Board of XuanWu Hospital, Capital Medical University, Beijing, China. Written informed consent was obtained from each participant.

The diagnosis of SVMCI was performed by two experienced neurologists in consensus according to criteria (Erkinjuntti et al., 2000; Román et al., 2002; Petersen, 2004; Moorhouse and Rockwood, 2008) that included the following: (1) subjective cognitive complaints reported by the participant or his/her caregiver; (2) objective cognitive impairments, although not meeting the Diagnostic and Statistical Manual of Mental Disorders, Fourth Edition (DSM-IV) criteria for dementia; (3) a Clinical Dementia Rating Scale (CDR) score = 0.5; (4) a Mini-Mental State Examination (MMSE) score ≥24 for middle school-educated, ≥20 for primary school-educated, and ≥17 for illiterate participants (Zhang et al., 1990); and (5) subcortical vascular causes of the cognitive impairments according to (a) moderate to severe WMH in at least one region with a Wahlund rating scale score ≥2 (Wahlund et al., 2001) and/or multiple lacunar infarcts in the periventricular and deep WM structures (Wahlund Rating Scale score ≥2; diameter, 15 mm) on T2-weighted or FLAIR images; and (b) evident neurological signs of hemiparesis, lower facial weakness, Babinski sign, dysarthria, sensory deficit, gait disorder, urgent urination, or motor slowness that were assessed by general and neurological examination or reported by the participant or his/her caregiver. The exclusion criteria for SVMCI included (Román et al., 2002; Zhou and Jia, 2009): (1) deficits in memory and other cognitive functions in the absence of focal lesions on brain imaging; (2) cognitive impairments as a result of other causes, such as tumor, epilepsy, traumatic brain injury, multiple sclerosis, psychiatric disease, systemic disease (e.g., thyroid dysfunction, severe anemia, syphilis, and HIV), lifetime alcohol or drug abuse; (3) a suffering of visual abnormalities, severe aphasia or palsy that made clinical assessments infeasible; (4) signs of large vessel diseases, such as cortical and/or corticosubcortical non-lacunar territorial infarcts and watershed infarcts or hemorrhages; and (5) diseases that led to WM lesions, such as normal pressure hydrocephalus, multiple sclerosis, sarcoidosis, or brain irradiation.

For aMCI patients inclusion, the Petersen criteria (Petersen, 2004) was fulfilled with the following modifications: (1) subjective memory complaint reported by the participant, preferably confirmed by his/her caregiver; (2) objective memory decline below 1.5 SD of the age- and education-adjusted norms on memory-related neuropsychological tests; and (3) the same diagnostic criteria as (3) to (5) in the above list for SVMCI. aMCI patients were excluded if they exhibited any of the following clinical characteristics: (1) severe depressive symptoms based on a Hamilton Depression Rating Scale score (Hamilton, 1960) > 24; (2) cognitive impairments caused by psychiatric disease, systemic disease (e.g., thyroid dysfunction, severe anemia, syphilis, or HIV), non-MCI neurological disorders, or lifetime alcohol or drug abuse; or (3) visual abnormalities, severe aphasia, or motor disorders that would render neuropsychological testing infeasible. Community residents of Beijing were recruited by an advertisement for the healthy control group. The healthy controls were not under any treatment and did not have any brain abnormalities or any history of neurological or psychiatric disorders and no cognitive complaints.

### Data Acquisition

All images were acquired using a 3.0 T Siemens Verio scanner at XuanWu Hospital, Capital Medical University. Structural images consisted of sagittal magnetization-prepared rapid gradient echo (MP-RAGE) T1-weighted and T2-weighted sequences. The T1-weighted sequence had the following parameters: repetition time (TR) = 1,900 ms; echo time (TE) = 2.2 ms; inversion time (TI) = 900 ms; flip angle (FA) = 9°; number of slices = 176; slice thickness = 1.0 mm; data matrix = 256 × 256; field of view (FOV) = 256 × 256 mm^2^. The T2-weighted images had the following parameters: TR = 4,040 ms; TE = 84 ms; FA = 160°; number of slices = 20; slice thickness = 5.0 mm; gap = 1.5 mm; data matrix = 320 × 186; FOV = 240 × 140 mm^2^. Resting-state functional images were acquired using an echo-planar imaging sequence, and acquisition parameters are: TR = 2,000 ms; TE = 40 ms; FA = 90°; number of slices = 28; slice thickness = 4 mm; gap = 1 mm; data matrix = 64 × 64; FOV = 256 × 256 mm^2^. The subjects were instructed to lie quietly in the scanner with their eyes closed and to remain stable as much as possible during the data acquisition. The functional scan lasted for 478 s (239 volumes) in total. During the scan, foam pads and headphones were used to reduce head motion and scanner noise as much as possible. The individuals were asked if they stayed awake after the scanning process was complete. Any individual who fell asleep was subsequently excluded from the study.

### Regions of Interest Selection

*A priori*-defined subdivisions of the BG were selected based on a fine-grained, cross-validated atlas of the human Brainnetome Atlas (Fan et al., 2016). The selected BG subdivisions included six bilateral regions of ventral caudate (vCa), dorsal caudate (dCa), globus pallidus (GP), nucleus accumbens (NAcc), ventromedial putamen (vmPu), and dorsolateral putamen (dlPu).

### WMH Quantification

We adopted an operator-driven quantitative approach to quantify WMH volume on T2-weighted images (Brickman et al., 2011). For each individual subject, we manually determined a WM intensity threshold that labeled voxels appearing as hyperintense by visual inspection of the T2-weighted image. WMHs of each subject were then extracted by application of a WM mask generated from the segmented T1-weighted anatomical image, and the percentage of WMH volume was calculated as a ratio of the number of hyperintense voxels over the total volume of the WM mask on the MRI images sampled. We also quantified the percentage of hyperintensities over BG structures by counting the number of hyperintense voxels (normalized by dividing the total number of voxels within the sampled region) located within each of the six bilateral BG nuclei preselected from the Brainnetome Atlas.

### Functional MRI Processing

Functional MRI images were preprocessed using the Analysis of Functional Neuroimaging software (Cox, 1996). The preprocessing steps consisted of slice timing correction, motion correction, spatial smoothing (FWMH = 6 mm), band-pass temporal filtering (0.01–0.1 Hz), spatial normalization to standard Talairach space and removal of the head motion profiles, the WM, and cerebrospinal fluid (CSF) signal. To moderate the effects of head motion on estimates of resting-state functional connectivity (rsFC) (Power et al., 2012; Satterthwaite et al., 2012; van Dijk et al., 2012), we first calculated the average root mean square (RMS) of head motion and found no significant between-group difference (*F* = 1.15, *P* = 0.32). The average RMS of head movement for all three groups was considerably below the cutoff of 1 mm (average RMS = 0.13 for NC, 0.13 for aMCI, and 0.14 for SVMCI). Second, we censored volumes within each subject’s fMRI time-series that were associated with sudden head movements. For each subject, fMRI volume was censored if it is frame-wise displacement (FD) > 0.35.

We computed rsFC of the BG regions as follows. Within each seed region, we calculated the Pearson correlations with Fisher’s *z*-transformation between the averaged time courses of the seed region and all other brain voxels. Within-group one-sample *t*-test was performed for each seed region, and an rsFC map for each group was created by applying a threshold of *p* < 0.0001 with a cluster size of 86 voxels (*P*_*corrected*_ < 0.001 based on Monte Carlo simulations).

### Statistical Analysis

Group differences in demographics were evaluated using one-way ANOVA followed by *post hoc* Tukey test for continuous variables and Chi–square test for categorical variables.

To examine the effect of group and seed regions on WMHs, we performed a two-way mixed-effect ANOVA of GROUP (SVMCI, aMCI, and NC) and SEED REGION (six bilateral BG regions), followed by *post hoc* Tukey tests in WMHs for each BG seed region.

To explore rsFC differences between SVMCI and NC groups across BG nuclei, we conducted a two-way mixed-effect ANOVA of GROUP (SVMCI versus NC) and SEED REGION (six bilateral BG regions) followed by *post hoc* two-sample *t*-tests. Similar ANOVA was also conducted to test differences in rsFC between aMCI and NC and between SVMCI and aMCI groups across BG subdivisions. To improve the sensitivity of detection while still controlling for the false-positive rate, a voxel-wise threshold of *P* < 0.01 combined with a cluster threshold of 60 voxels (*P*_*corrected*_ < 0.05) as derived from Monte Carlo simulation was used to obtain the group difference map, with the restriction that significant clusters must belong to the “OR” rsFC map of the six bilateral seed regions in at least one group.

In order to explore to which functional brain systems the regions targeted by MCI (i.e., the regions showing significant ANOVA results) belong, we conducted a graph-theory-based modularity analysis on averaged whole-brain network from the NC group to decompose brain modules that correspond to known functional systems (see Supplementary Text for details of the modularity analysis). Brain regions from the ANOVA were overlapped with the resultant modular structure to identify their functional affiliations.

### Associations Among WMH, Functional Connectivity, and Behavioral Performance

To investigate WMHs in which BG regions would correlate with clinical/behavioral performance, we conducted a least absolute shrinkage and selection operator (LASSO) regression analysis using GLMNET toolbox in R to select candidate predictors of WMH in all six bilateral BG nuclei to predict the dependent variable of MMSE and the three AVLT subscale scores, respectively. LASSO is a modified form of least squares regression that penalizes complex models with a regularization parameter (λ) (Tibshirani, 2011). GLMNET uses cross-validation to identify the optimal regularization term λ that would minimize the mean cross-validated error for the fitted model. For our analysis, we used a *k* = 10-fold cross-validation approach for LASSO model fit. We also performed LASSO regression analyses to explore relationships between clinical/behavioral scores and the BG functional connectivity affected by MCI disease (i.e., those showing significant effects in the above ANOVA). To reduce the dimension of predictive variables and promote the regression fitting effect, averaged rsFCs from the MCI-affected regions that belong to the same functional system were used as independent variables in the LASSO regression model. For each BG seed region, we also evaluated the correlations between WMH in each BG region and its MCI-affected rsFC. Note that for each BG region of interest (ROI), its rsFCs were also averaged across the MCI-affected regions that belong to the same functional system.

## Results

### Demography and Behavior

Table 1 shows the demographic data of the healthy controls, the patients with aMCI, and the patients with SVMCI. All groups were well-matched in terms of age[(*F*(2,117) = 0.025, *P* = 0.97], gender (χ^2^ = 0.625, *P* = 0.73), and years of education (*F*(2,117) = 1.14, *P* = 0.32). Moreover, as revealed by one-way ANOVA, there were significant group differences in MMSE [*F*(2,117) = 19.7, *P* < 0.001], AVLT-immediate [*F*(2,117) = 32.6, *P* < 0.001], AVLT-delayed recall [*F*(2,117) = 48.1, *P* < 0.001], and AVLT-recognition scores [*F*(2,117) = 22.4, *P* < 0.001]. *Post hoc* tests revealed that all of the AVLT subscale scores were significantly lower in the aMCI group compared with those of the SVMCI and NC groups (*AVLT subscale scores: P_*s*_* < 0.001). Patients with aMCI were also more impaired in MMSE (*P* = 0.02) than those with SVMCI. To control for potential confounding effects of the MMSE difference between the two MCI subgroups, we repeated our main analyses using a subgroup of the aMCI patients whose MMSE scores matched with the SVMCI groups (SVMCI: *n* = 40; aMCI: *n* = 29, *P* = 0.78) and validated if the differences between MCI groups represent effects solely by MCI subtype.

**TABLE 1:** Demographic characteristics of the participants.

	**NC (*n* = 40)**	**aMCI (*n*** = **40)**	**SVMCI (*n* = 40)**	***P*-value**
Gender (M/F)	15/25	15/25	18/22	0.73
Age	64.67 ± 5.8	64.85 ± 10.8	64.4 ± 9.3	0.97
Education years	10.6 ± 5.25	9.6 ± 4.5	8.975 ± 4.75	0.32
MMSE	28.2 ± 2.1	24.725 ± 3.7	25.575 ± 3.6	<0.001^∗^
AVLT-Immediate recall	9.075 ± 1.6	5.6 ± 1.4	7 ± 2.6	<0.001^∗^
AVLT-Delayed recall	9.925 ± 2.6	3.65 ± 2.9	7.025 ± 3.07	<0.001^∗^
AVLT-Recognition	11.975 ± 2.7	7.325 ± 4.05	10.5 ± 2.5	<0.001^∗^

*^∗^A significant difference across the three groups. *N*, number of subjects; NC, normal control; aMCI, amnestic mild cognitive impairment; SVMCI, subcortical vascular mild cognitive impairment group; MMSE, Mini-Mental State Examination; AVLT, Auditory Verbal Learning Test. Means ± standard deviation.*

### BG Hyperintensities Are Heterogeneously Distributed Across Different Subdivisions

WMHs were found to be present in subjects with or without SVMCI or aMCI, but their volume and anatomical distribution exhibited considerable variations between groups as well as across different brain structures. One-way ANOVA showed a significant group effect of WM hyperintensities [*F*(2, 105) = 15.45, *P* < 0.001]. Patients with SVMCI exhibited more WMHs than patients with aMCI (*P* < 0.001) and healthy controls (*P* < 0.001). There was no significant difference between healthy controls and aMCI group (*P* = 0.10).

**FIGURE 1:**
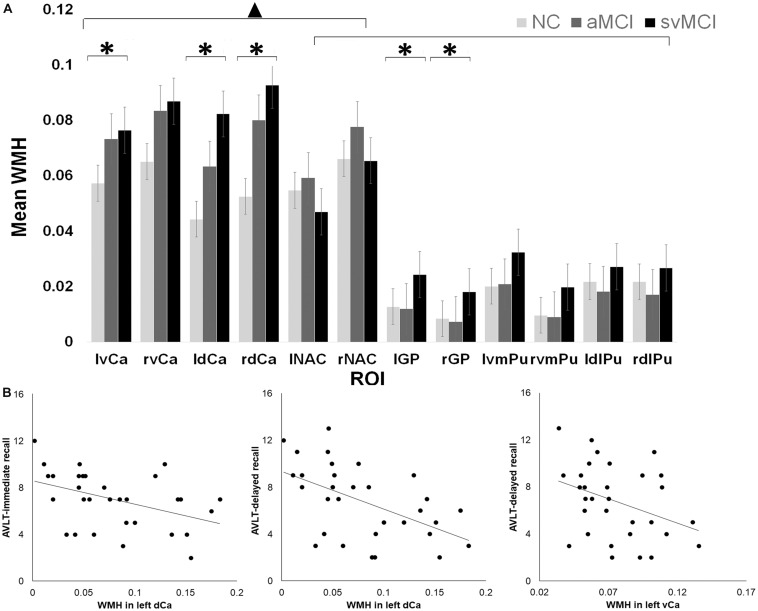
**(A)** White matter hyperintensity (WMH) differences across different groups and regions of interest (ROI). ▲ indicates a significant difference between caudate and the other basal ganglia (BG) regions. ^∗^ indicates significant group by ROI effect in certain regions. **(B)** Relationship between Auditory Verbal Learning Test (AVLT) scores and WMH in left dorsal and ventral caudate in subcortical vascular mild cognitive impairment (SVMCI) patients.

Hyperintensities within each BG nucleus showed significant group by ROI effect as determined by ANOVA [*F*(22, 1260) = 1.86, *P* = 0.009; Figure 1A]. While significant group differences were observed for bilateral GP [left: *F*(2,105) = 5.35, *P* = 0.006; right: *F*(2,105) = 4.12, *P* = 0.02], dCa [left: *F*(2,105) = 5.69, *P* = 0.005]; right: [*F*(2,105) = 4.64, *P* = 0.01)], and left vCa [*F*(2,105) = 4.10, *P* = 0.02], there was no significant group difference for the other BG structures (*P*_*s*_ > 0.05). Interestingly, in all three groups, WM lesions were not evenly distributed across different BG subdivisions as indicated by a significant ROI effect in WMH [*F*(11, 1260) = 49.7, *P* < 0.001; Figure 1A]. Tukey *post hoc* tests found a remarkably higher percentage of volume of hyperintensities in dCa and vCa than in the other BG regions (*P*_*s*_ < 0.05), which suggests that the caudate areas might be more vulnerable to WM impairments associated with normal or neurodegenerative aging.

To investigate the relationship between the behavioral metrics and the amount of WMH, we performed LASSO regression to select candidate predictors (WMHs and hyperintensities over BG nuclei) of verbal learning assessment scores. For the SVMCI group (Figure 1B), LASSO analysis selected hyperintensities over WM (LASSO coefficient: −10.11) and the left dCa (LASSO coefficient: −5.68) as significant predictors for declined AVLT-immediate recall performance (explained 21% of the variance in AVLT-immediate recall performance), while WMHs within the left ventral (LASSO coefficient: −1.94) and dCa (LASSO coefficient: −5.50), on the other hand, were found to be predictive of worse performance in AVLT-delayed recall (explained 17% of the variance in AVLT-immediate recall performance). In contrast, none of the WM impairments were selected to be able to predict verbal learning performance in aMCI patients. Validation analysis performed in a subset of aMCI patients whose MMSE scores matched with the SVMCI group showed consistent results.

### Functional Network Connectivity of BG Nucleus Is Altered Differently in aMCI and SVMCI Patients

Generally, all three groups showed similar spatial topography patterns of rsFC with each of the six bilateral BG regions (Supplementary Figure S1), which are similar to previously described patterns.

**FIGURE 2:**
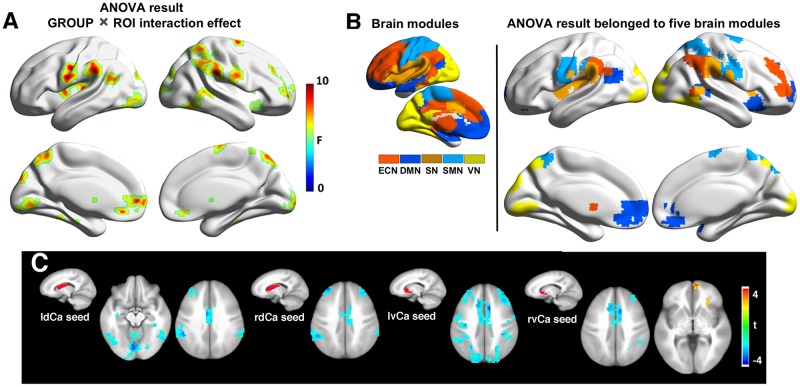
**(A)** Group-regions of interest (ROI) interaction effects in basal ganglia (BG) functional connectivity between subcortical vascular mild cognitive impairment (SVMCI) and normal control (NC) groups. **(B)** Brain regions with significant group (SVMCI vs. NC) by ROI interaction effects are distributed in five major brain systems. **(C)** Brain regions showing significant differences between SVMCI and NC groups in functional connectivity with dorsal and ventral caudate nucleus. The brain surface maps were rendered by using the BrainNet Viewer (http://www.nitrc.org/projects/bnv/) (Xia et al., 2013).

Mixed ANOVA discovered a significant group by seed region interaction effect between SVMCI and NC groups in extensive areas, including predominantly the medial and orbital prefrontal gyrus, bilateral dorsolateral prefrontal gyrus, inferior and superior parietal gyrus (Figure 2A). We next considered the functional affiliation of these regions in the context of modular structure derived from the NC group. Five major functional brain modules were found: the default mode network (DMN), the executive control network (ECN), the salience network (SN), the sensorimotor network (SMN), and the visual network (VN) (Figure 2B). Based on the identified modular structure, the targeted regions in SVMCI belonged primarily to the ECN (including mainly the bilateral dorsolateral frontal and parietal regions), followed by the DMN (including mainly the medial orbitofrontal regions), SMN (including the precentral and postcentral areas), VN (including regions of cuneus and fusiform), and SN (including the insular and superior temporal areas).

*Post hoc t*-tests revealed that, of the six BG nuclei, only the dCa and vCa exhibited significant abnormal rsFC in patients with SVMCI. Specifically, compared with NC, both the dCa and vCa showed significantly weaker rsFC to a range of ECN regions in SVMCI (Figure 2C), including the dorsal anterior cingulate cortex, the bilateral dLPFC, and parietal cortices. Furthermore, patients with SVMCI showed significantly higher rsFC between the right vCa and the rostral anterior cingulate cortex, which is a key region of the DMN network.

**FIGURE 3:**
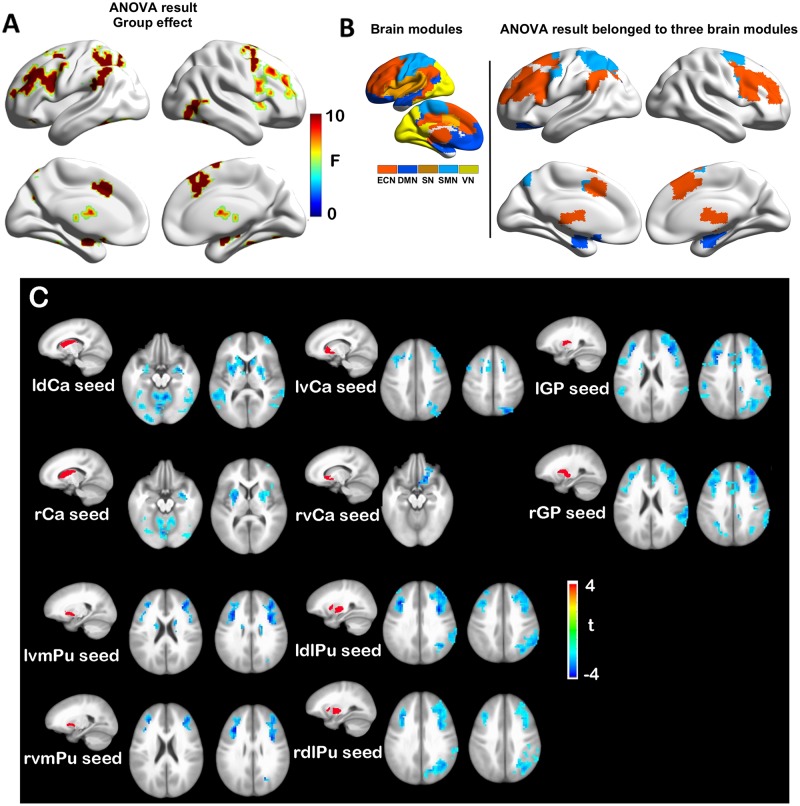
**(A)** Group effects in basal ganglia functional connectivity between amnestic mild cognitive impairment (aMCI) and normal control (NC) groups. **(B)** Brain regions with significant group (aMCI vs. NC) effects are distributed in three major brain modules. **(C)** Functional connectivity differences between aMCI and NC groups with each basal ganglia seed region.

In contrast to functional dysconnectivity in specific BG subdivisions in SVMCI, significant group effects were observed between aMCI and NC group across seed regions, indicating broader dysregulation of BG rsFC in aMCI. Brain regions with significant group effects were distributed primarily in bilateral frontal and superior temporal cortices, as well as the hippocampus/parahippocampus (Figure 3A), which belonged to three functional modules: the ECN (including mainly the dorsal anterior cingulate, the bilateral dorsolateral frontal and parietal regions, and the thalamus), DMN (including mainly the bilateral hippocampus/parahippocampus), and SMN (including regions in postcentral gyrus) (Figure 3B). *Post hoc* analyses observed that (Figure 3C), while most of the BG nuclei exhibited a similar dysconnectivity pattern, demonstrating connectivity decline in areas such as frontal, temporal, and parietal cortices, the NAcc seed showed relatively fewer connectivity differences. Moreover, weaker connectivity with the hippocampus/parahippocampus area was observed to be most prominent in bilateral dCa subdivisions.

Mixed ANOVA directly contrasting between the SVMCI and aMCI groups found significant group effects primarily in the regions of prefrontal and temporal cortices. *Post hoc* comparisons between aMCI and SVMCI revealed that, while the vCa and NAcc regions showed none or few group differences, the other BG nuclei exhibited significantly weaker functional connectivity with regions across the ECN system in aMCI patients (Supplementary Figure S2). Similar analysis performed on a subset of data also showed group differences between aMCI and SVMCI group to be primarily in the regions of prefrontal and temporal cortices (Supplementary Figure S3).

**FIGURE 4:**
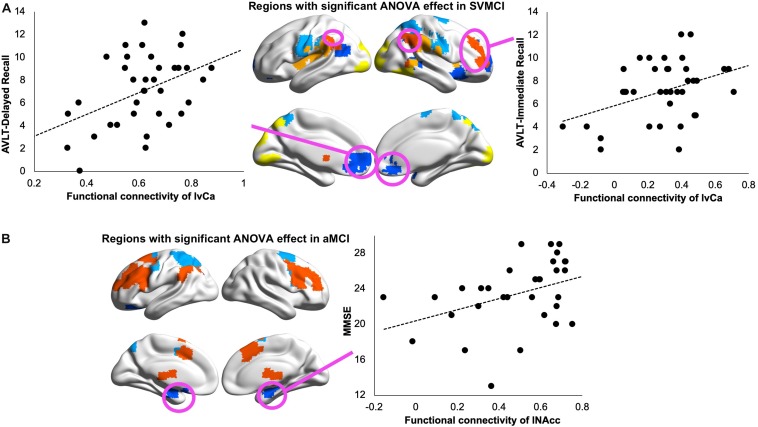
**(A)** Relationship between Auditory Verbal Learning Test (AVLT) immediate recall scores and resting-state functional connectivity (rsFC) of left ventral caudate with subcortical vascular mild cognitive impairment (SVMCI)-affected regions belonging to the executive control network (ECN; left panel) and between AVLT delayed recall scores and rsFC of left ventral caudate with SVMCI-affected regions belonging to the default mode network (DMN; right panel) in SVMCI patients. **(B)** Relationship between Mini-Mental State Examination (MMSE) and rsFC of left nucleus accumbens (NAcc) with amnestic MCI (aMCI)-affected regions belonging to the DMN in aMCI patients.

We performed LASSO regression to understand the effect of altered BG connectivity on learning and memory. In SVMCI patients, LASSO regression selected the disease-affected rsFC between the left vCa and regions that belong to the ECN as a significant predictor for AVLT-immediate recall performance (LASSO coefficient: 0.57, explained 15% of the variance in AVLT-immediate recall performance; Figure 4A) and the rsFC between the left vCa and the regions that belong to the DMN (LASSO coefficient: 2.33) as a significant predictor for AVLT-delayed recall performance (explained 22.6% of the variance in AVLT-delayed recall performance; Figure 4A). As for the aMCI group (Figure 4B), the only significant relationship was observed between MMSE scores and the disease-affected rsFC between the left NAcc and the regions belonging to the DMN (LASSO coefficient: 2.32, explained 17.7% of the variance in MMSE). Similar results were observed in the validation analysis as well.

### Associations Between WMHs and BG Functional Connectivity

**FIGURE 5:**
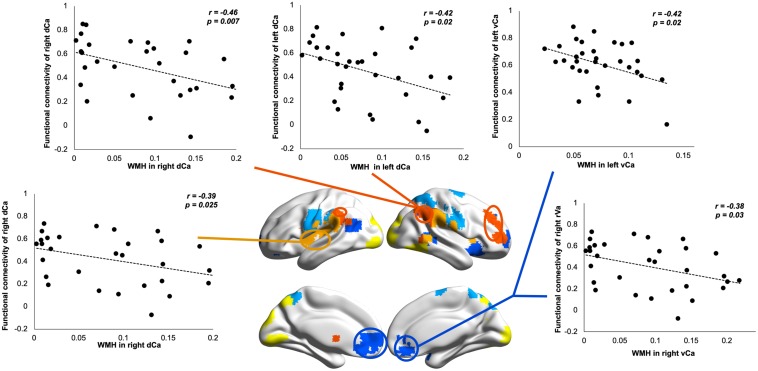
Relationship between white matter hyperintensity (WMH) in dorsal/ventral caudate and their resting-state functional connectivity (rsFC) in subcortical vascular mild cognitive impairment (SVMCI) patients.

To understand how WM impairments impact degenerated BG functional connectivity, we correlated WMHs in each BG nucleus with its functional connectivity showing significant group differences. Our results revealed that, in SVMCI patients, only WMH impairments in the caudate nucleus were related to functional dysconnectivity with cortical networks, especially those of significant associations with AVLT memory performance (Figure 5). Specifically, WMHs in the bilateral dCa correlated negatively with their disease-affected connectivity with the regions belonging to the ECN (left dCa: *r* = −0.42, *P* = 0.02; right dCa: *r* = −0.46, *P* = 0.007) and SN (right dCa: *r* = −0.39, *P* = 0.025), while WMHs in the vCa correlated negatively with their connectivity with the regions in the DMN (left vCa: *r* = −0.42, *P* = 0.02; right vCa: *r* = −0.38, *P* = 0.03). In contrast, in aMCI patients, there was no relationship between WMH impairments in any of the BG regions with their functional connectivity. Similar results were also observed in the subgroup of aMCI patients matching MMSE scores with the SVMCI group.

## Discussion

The current study revealed that, although both aMCI and SVMCI patients appeared to have greater WMHs, SVMCI patients are characterized by more severe WMH impairments in BG areas. WMHs in both SVMCI and aMCI groups were distributed heterogeneously across different BG subdivisions, with the greatest hyperintensities observed in the dCa and vCa regions. Importantly, such anatomical WMH heterogeneity across BG regions was associated with changes in their rsFC and verbal memory performance only in patients with SVMCI.

A key finding from this study is the heterogeneously distributed WMHs across different BG nucleus in both SVMCI and aMCI patients. WMHs mostly reflects demyelination and axonal loss as a consequence of SVD (Prins and Scheltens, 2015). A characteristic feature of the cerebral small vessel system is its marked anatomical and functional heterogeneity. Within the BG, the architecture of arterioles and capillaries, the most affected small vessel types in SVD (Okeda et al., 2004; Brown et al., 2007; Onodera et al., 2015; Akashi et al., 2017), differs notably in different nuclei (Nonaka et al., 2007). Previous evidence has demonstrated that perforating arterioles running within the BG have rich small branches only in the caudate and putamen nucleus (Nonaka et al., 2007). Moreover, the caudate nucleus is also noted by the denser capillary network compared with that in other BG structures (Nonaka et al., 2007). Therefore, it appears that the microvasculature architecture of the caudate nucleus is characterized by affluent perforating arterioles and capillaries. This may serve as a possible explanation for the most substantial WMHs observed in dCa and vCa, although further study is warranted to better elucidate the differential vulnerability of BG structures to WM lesions caused by cerebral SVD.

Although both SVMCI and aMCI patients showed similar WM lesion patterns, the two groups were found to differ from each other in terms of BG rsFC changes, as well as their relationships with WMH alterations. In SVMCI patients, of all the BG nuclei, only the dCa and vCa were found to show significant rsFC changes, which were closely correlated with WM lesions within the corresponding BG regions. By comparison, rsFC changes in aMCI patients were not restricted to specific BG nucleus but were instead more extended in almost all BG areas. More importantly, there was no significant correlation between aMCI-related dysconnectivity and WMHs in any of the BG nucleus. These findings suggest a differential involvement of cerebrovascular disease pathology in driving dysregulated BG rsFC between SVMCI and aMCI patients. Previous studies have demonstrated that AD is associated with the enlargement of perivascular spaces (PVS) in the centrum semi-ovale, but not the severity of PVS in BG, which is instead associated with subcortical vascular cognitive impairment (SVCI), suggesting that these two clinical subtypes of cognitive impairment could be characterized by distinctively distributed neuroimaging markers of SVD associated with different underlying pathology (Banerjee et al., 2017). Our results are in accord with these lines of research and, of more significance, provide evidence establishing a link between small vessel pathology in BG and deficiencies of its functional network integrity in SVMCI.

An interesting finding in the SVMCI group is that, other than the hypoconnectivity found with the dCa, the vCa showed higher FC with the rostral anterior cingulate, indicating opponent modulation effect involved in discrete striato-cortical circuits along the dorsal-ventral axis. Previous studies have established that different parts of the striatum are differentially enhanced or suppressed by serotonergic modulation (Tanaka et al., 2007; Schweighofer et al., 2008). Specially, under low serotonin levels, the activity of the ventral striatum appeared to be elevated while the activity in the dorsal striatum was depressed (Tanaka et al., 2007), which coincides with the bidirectional striatal FC changes in SVMCI. Indeed, neurochemical investigation of brain tissue from patients with vascular dementia has demonstrated that the serotonin metabolism is severely reduced in caudate areas (Gottfries et al., 1994). Therefore, SVD-related reductions of serotonin metabolism may differentially modulate the circuits of cortical to dorsal and ventral striatum. Future research could be done to probe the serotonin signaling origins of these connectivity changes and examine how these changes causally contribute to deficits in episodic learning and memory.

Our findings of weaker functional coupling between BG and the regions in the ECN in aMCI are consistent with previous cross-sectional reports of decreased subcortical-frontal functional coupling in aMCI (Bai et al., 2011) and decreased ECN integrity in both aMCI and AD patients (Chong et al., 2017). Interestingly, these rsFC changes were not related to WM lesions in aMCI, which indicates that there might be different mechanisms other than WMH pathologies underlying the dysregulations of BG rsFC in aMCI. Although hippocampus/medial temporal structures have been considered as primary loci responsible for cognitive dysfunctions in AD, recent studies have begun to establish a link between deficits in dopaminergic BG nucleus and the cognitive symptoms of AD (Trillo et al., 2013), which suggests the involvement of the dopaminergic system in the pathophysiology of the disease. Key findings in this context include the observation that profound morphological and biochemical changes in BG nucleus were accompanied by amyloid deposition in transgenic AD mice (Perez, 2005). Pharmacological treatments that restore dopaminergic transmission decreased intraneuronal Aβ accumulation and improved learning and memory in animal models of AD, suggesting that the dopaminergic, amyloid, and cognitive signatures of AD are causally related (Ambrée et al., 2009; Eri et al., 2010; Guzman-Ramos et al., 2012). Furthermore, studies in AD patients have observed markedly decreased expression of dopaminergic receptors in distributed brain regions (Kemppainen et al., 2003; Kumar and Patel, 2007). Taken together, these findings raise the possibility that the changes in BG rsFC documented here may comprise network-wise consequences of distributed dopaminergic dysfunctions interfered by prolonged Aβ exposure. The testable prediction based on this proposal is that interventions that normalize dopaminergic and Aβ pathologies will rescue BG rsFC in aMCI or AD model.

There remain several limitations in this study. As a cross-sectional design, our study was unable to disentangle the causal relationship between WM impairments and functional network dysconnectivity. Future longitudinal studies would be better equipped to track changes in these measures as the disease progresses. Furthermore, the spatial resolution of T2-weighted images in the current study is relatively low by today’s standards, which may potentially impede accurate estimation of WMH in BG nuclei. Future studies using T2 or FLAIR images with better spatial resolution are needed to validate our current findings. Moreover, although the present study revealed altered striatal networks and their relationship with verbal learning and memory in SVMCI, task-based fMRI studies may help to better understand the neural mechanism underlying these relationships. Finally, patients with MCI commonly suffer from depression and other major affective conditions. Similarly, frequent reports of cognitive abnormalities are also seen in depressed individuals. Clinical variables such as a shorter duration of untreated depression have been shown to be associated with more favorable treatment outcomes (Ghio et al., 2015). Future studies investigating brain network dysconnectivity in patients with MCI as well as major affective disorders may help to understand the pathological underpinnings of dysregulated neural circuits and their impact on clinical outcomes of cognitive and affective impairment comorbidity.

## Conclusion

In conclusion, our results reveal that the WMHs are distributed heterogeneously across different BG regions in both MCI subtypes, demonstrating that the caudate nuclei are among the structures most vulnerable to cerebrovascular events during neurodegeneration. In SVMCI, WMHs in caudate along the dorsal-ventral axis are characterized by functional dysconnectivity of discrete striatal-cortical circuits, establishing a link between cerebrovascular-related structural abnormality, integrity of BG circuits, and episodic memory impairments in SVMCI. In contrast, in aMCI patients, we failed to observe any significant relationship between WMHs in BG regions and their functional connectivity. These findings suggest a differential role that the cerebrovascular pathology played in disrupting network-level communications in these two MCI groups and may provide insights in developing novel imaging biomarkers that improve our ability to discriminate between Alzheimer’s and subcortical vascular dementia.

## Data Availability Statement

The datasets generated for this study are available on request to the corresponding author.

## Ethics Statement

The studies involving human participants were reviewed and approved by the Medical Research Ethics Committee and Institutional Review Board of Xuanwu Hospital, Capital Medical University, Beijing, China. The patients/participants provided their written informed consent to participate in this study. Written informed consent was obtained from the individual(s) for the publication of any potentially identifiable images or data included in this article.

## Author Contributions

AA, XL, YH, and LY designed the research. AA, XL, and LY performed the research. AA and XL analyzed the data. AA, XL, WT, CJ, YH, and LY wrote the manuscript.

## Conflict of Interest

The authors declare that the research was conducted in the absence of any commercial or financial relationships that could be construed as a potential conflict of interest.
