# Supplementary material for: White Matter Hyperintensities Relate to Basal Ganglia Functional Connectivity and Memory Performance in aMCI and SVMCI
Source: Front Neurosci. 2019 Nov 13;13:1204. doi: 10.3389/fnins.2019.01204 (PMC6874172; doi:10.3389/fnins.2019.01204)
Supplement: Supplementary file 1 [file Table_1.DOCX]

**SUPPLEMENTAL MATERIAL**

**White Matter Hyperintensities Relate to Basal Ganglia Functional Connectivity and Memory Performance in aMCI and svMCI**

**SUPPLEMENTARY TEXT**

In order to explore to which functional brain systems the regions targeted by MCI (i.e. the regions showing significant ANOVA results) belong, we conducted a graph-theory based modularity analysis on averaged whole brain network from the NC group to identify the architecture of brain modules that correspond to known functional systems. Modularity analysis allows to topologically divide nodes in a network (e.g. regions in the brain) into modules (Newman, 2006). Modules are groups of nodes that highly connected within its own module but less connected with other modules. In the current study, we defined network nodes by parcellating the brain into regions based on the Brainnetome atlas. Pearson correlation with Fisher's z-transformation was calculated between any pair of the network nodes to measure interregional functional connectivity. A group-averaged brain network was obtained by averaging functional correlation matrices across the NC subjects and thresholded by retaining the strongest 10% interregional correlations. The group-averaged brain network was then subjected to modularity analysis using the Louvain Algorithm (Blondel et al., 2008) .

**References**

Newman, M.E.J., 2006. Modularity and community structure in networks. Proc. Natl. Acad. Sci. 103, 8577–8582.

Blondel, V., Guillaume, J.-L., Lambiotte, R., Lefebvre, E., 2008. Fast unfolding of communities in large networks. Journal of Statistical Mechanics: Theory and Experiment P10008

**SUPPLEMENTARY FIGURE**


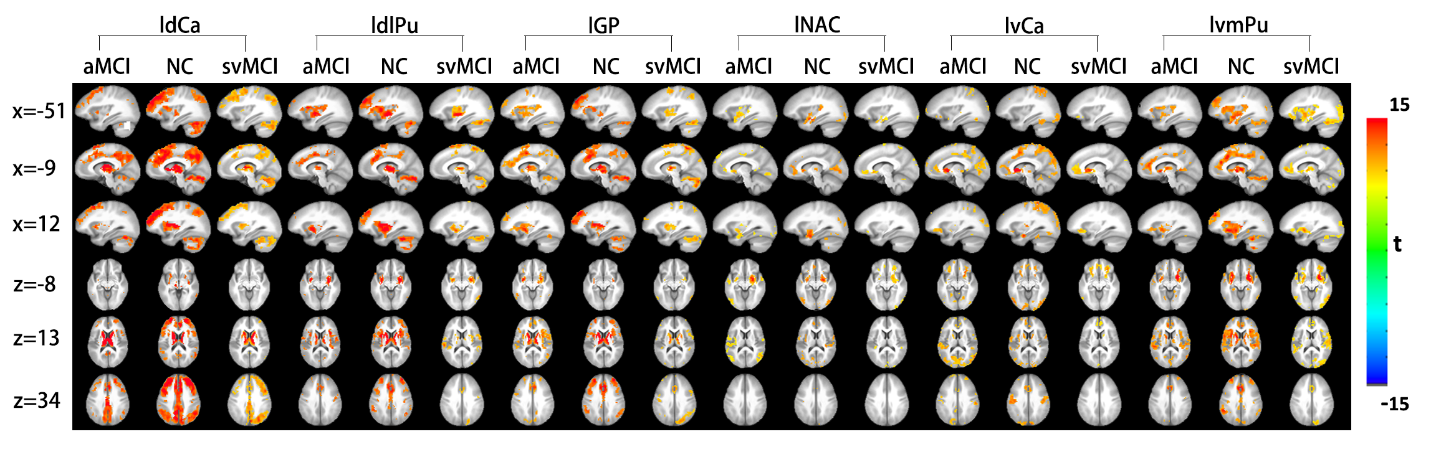


Supplementary Fig. 1. Functional Connectivity map of Basal Ganglia Nucleus in NC, aMCI and svMCI groups


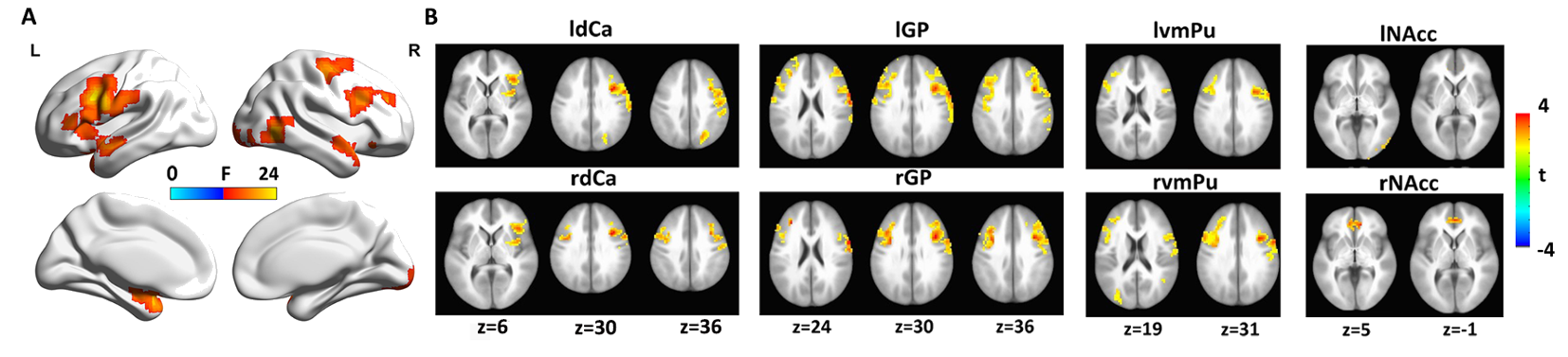


Supplementary Fig. 2. (A) Group effects in basal ganglia functional connectivity between aMCI and svMCI groups. (B) Functional connectivity differences between aMCI and svMCI groups with each basal ganglia seed region.


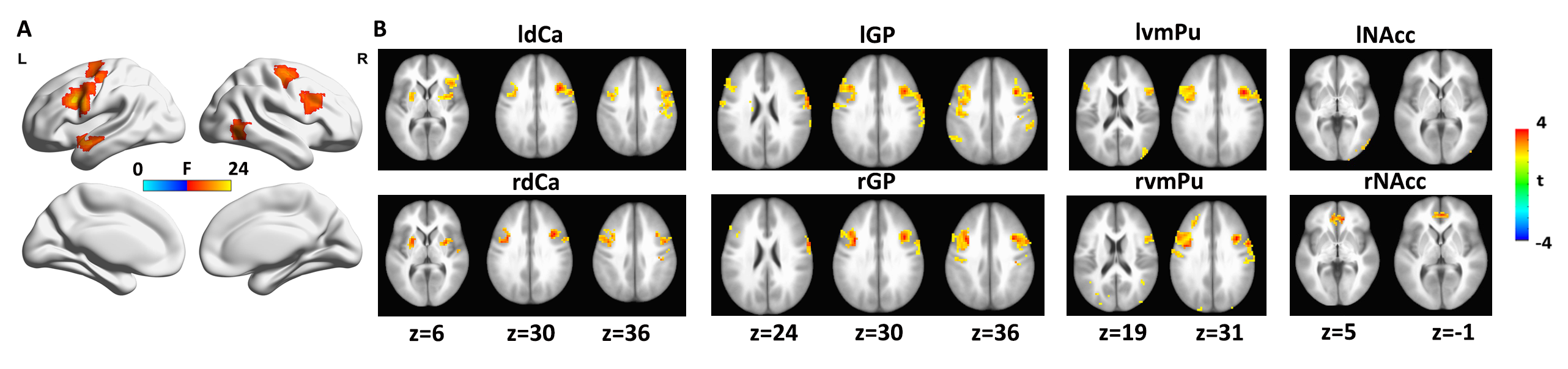


Supplementary Fig. 3. (A) Group effects in basal ganglia functional connectivity between aMCI and svMCI groups in MMSE matched subset of data. (B) Functional connectivity differences between aMCI and svMCI groups with each basal ganglia seed region in MMSE matched subset of data.
